# Measuring inequality in eye care: the first step towards change

**Published:** 2016

**Authors:** Jacqui Ramke

**Affiliations:** University of New South Wales, School of Social Sciences, Sydney, New South Wales, Australia.

**Figure:**
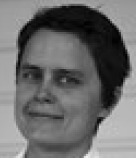
Jacqui Ramke

‘Health inequalities’ are differences in health between different subgroups of a population^1^, for example women/men, people with/without disabilities, and urban/rural dwellers.

Many of us have insufficient information to understand the nature and extent of the inequalities that exist, and whether our services are effective. This lack of information restricts our ability to plan appropriate strategies to reduce inequality, and to track our progress towards equitable eye health.

Fortunately, we can obtain this information by monitoring health inequality. Monitoring is a process that helps to determine whether policies and practices are working, and whether change is needed. There are two main sources of data we can use to monitor inequality-population-based surveys, and information collected from our clinics. Ideally, we would use information from both of these data sources. However, few of us have the time and money to implement population-based surveys, so this article will focus on monitoring inequality using clinic-based data. For example, you may have noticed that, compared to the community served by your hospital, most of the people undergoing cataract surgery are from the families of government employees (and very few are farmers) or from a ‘wealthy’ area in town (and very few from poorer areas, or rural areas), or from the most powerful ethnic, religious, or language group (and very few from minority groups). Or perhaps you have noticed that very few of your surgical patients are elderly widows. Collecting clinic-based information is a way to confirm or uncover these sorts of inequalities.

## Who should we monitor?

To reduce inequality, we must identify which **subgroup(s)** of the population (e.g. farmers, people from poorer or urban areas, or minority groups) are less able to get access to, and benefit from, our services. Some of us work in settings where the Ministry of Health and/or hospital has already identified priority subgroups to monitor, so advice and resources may be available locally. For others, we will need to decide which subgroups are most relevant to monitor in our particular setting.

**Figure:**
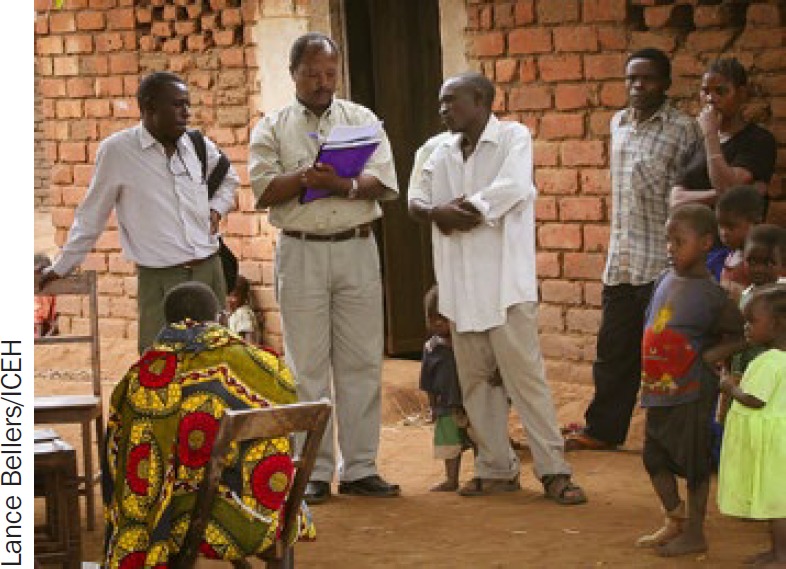
Gathering appropriate information helps us to understand inequality. TANZANIA

**Figure 1.**
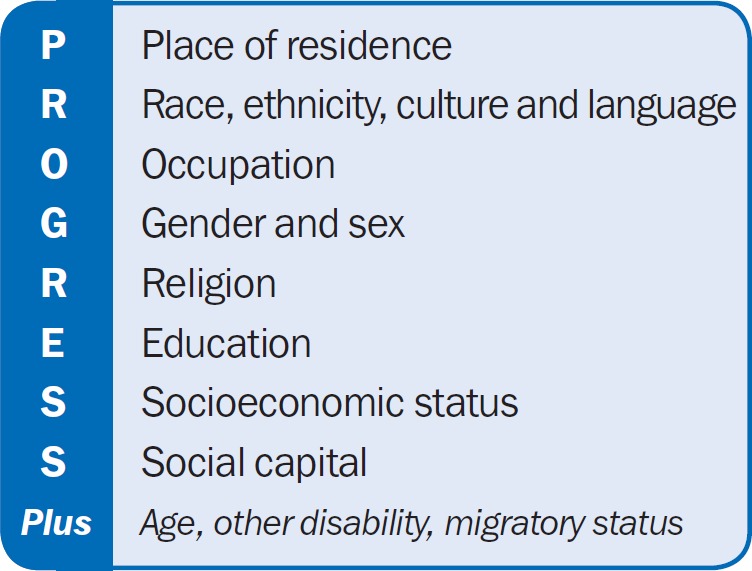
PROGRESS: social factors associated with health inequality^2^

The acronym ‘PROGRESS’ can help us to think about which subgroups to monitor, as it sets out a range of social factors that are often associated with health inequality (Figure 1.)^2^ Some of these have obvious subgroup categories (e.g. age, gender, disability), but others require us to adopt clear and consistent definitions, e.g. socioeconomic status, education level, area of residence (rural vs urban) or occupation category.

## What should we monitor?

Once we have identified the subgroups, we can use any of our routinely collected **health indicator(s)** to investigate inequalities between the subgroups. These indicators include:

- The prevalence of conditions (e.g. blindness, visual impairment, trachomatous trichiasis, diabetic retinopathy)
- Quality of care (e.g. visual outcome after cataract surgery).
- Access to services by different groups; e.g. cataract surgical coverage, cataract surgical rate, refractive error correction coverage, attendance at diabetic retinopathy screening, ability to pay
- Eye care service factors; e.g. the distribution of eye care facilities and the eye health workforce, and the availability of subsidised services or financial protection for vulnerable subgroups.

## Equality vs equity

We must remember that equal rates of treatment between subgroups (such as surgery) will not necessarily mean we are delivering equitable services. For example, the Nigerian National Blindness and Visual Impairment Survey showed that, although women had received almost half of all cataract surgery (47%), they still suffered from two-thirds of the bilateral cataract blindness (67%)^4^ in the country. This means that Nigerian women must receive **much more than 50%** of all the operations in order to reduce this inequity.

## How do we monitor inequality?

**Figure 2:**
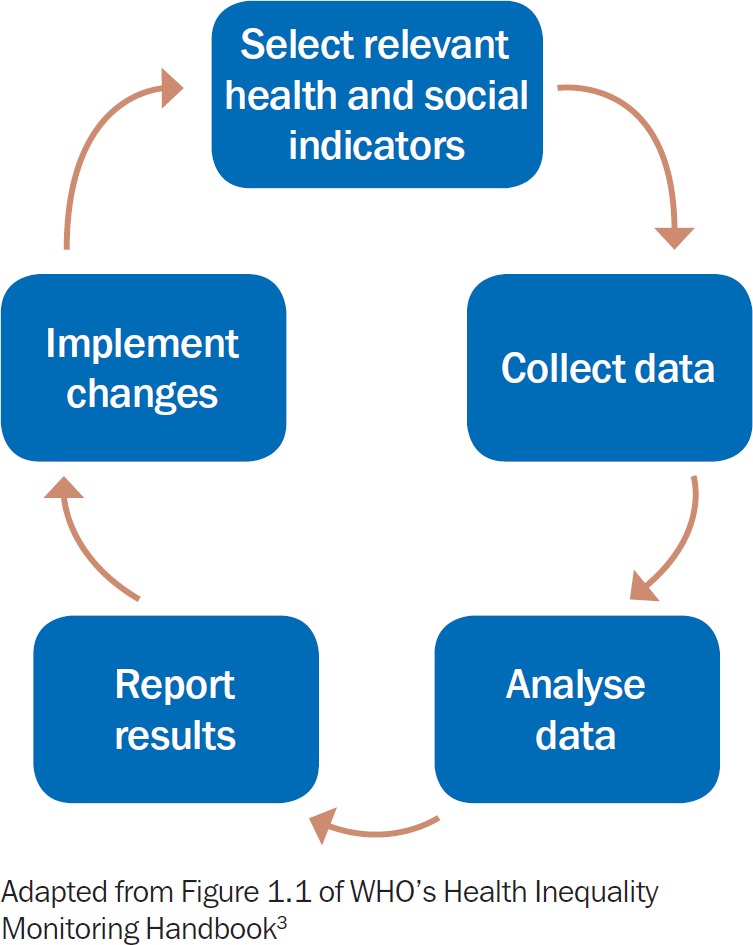
Cycle of health monitoring

Adapted from Figure 1.1 of WHO's Health Inequality Monitoring Handbook^3^

We can incorporate inequality monitoring into our hospital or clinic's existing system, whether electronic or manual. The monitoring cycle is shown in Figure 2.

We have already selected the relevant health and social indicators and chosen the subgroups we want to monitor. The next step is to collect the data. If we want to know if there is inequality in who receives cataract surgery, for example, we can begin to record, on a regular basis (e.g. monthly), the number of people in each of our selected subgroups who are receiving surgery.

Once we have collected the data, we can calculate, and then compare, the proportion of cataract surgery delivered to each subgroup (e.g. by dividing the number of women by the total number of operations). These figures are often presented as percentages (the proportion multiplied by 100). Another simple way to quantify inequality is to calculate the gap between the subgroups (e.g. subtract the number of women from the number of men to see how many more men have received surgery).

This can be done on a monthly, quarterly and annual basis, and inequality calculations can be reported alongside the total number of operations in each subgroup in well-designed tables, graphs and maps. The information can then be communicated to hospital administrators and health managers. A worked example is provided below.

When expanding your monitoring process, try to be realistic about what is feasible and sustainable in your setting. It is better to begin with a small number of indicators (such as uptake of cataract surgery by gender and urban/rural residence) and collect and analyse these accurately and consistently, rather than introducing many measurements that take a lot of time and effort; which means it will become unsustainable. You can expand your monitoring system with more indicators once it is running smoothly.

Monitoring is essential if we are to understand the nature and extent of inequality in the populations we serve. The information must then be used to inform policies, programmes and practices to reduce inequities and ultimately achieve universal eye health. *For more information on monitoring inequality, see WHO'S Health Inequality Monitoring Handbook:* **http://www.who.int/gho/health_equity/handbook/en/**
