# Supplementary material for: Measuring inequality in eye care: the first step towards change
Source: Community Eye Health. 2016;29(93):6–7. (PMC4995833)
Supplement: Related texts

# Brief report: Worked example

**Published:** 2016

Imagine your eye clinic is in an urban centre (population 150,000) that also serves the surrounding rural district (population 350,000). You conduct intermittent outreach services, and would like to conduct more as you think few rural dwellers are coming to your eye clinic.

You begin to monitor who is presenting for cataract surgery. In the first quarter you conduct no outreach trips and your monitoring data for January to March is shown in Table 1.

**Table 1.** Tally of cataract operations by gender and area of residence

Number of operations			
	Women	Men	Total
**Rural**	4	31	35
**Urban**	91	94	185
**Total**	95	125	220

You can work out and compare what percentage of the total number of operations were performed on patients belonging to each subgroup, e.g.:

- 57% of patients (125 ÷ 220 × 100) were men
- 43% of patients (95 ÷ 220 × 100) were women
- 84% of patients (195 ÷ 220 × 100) were urban
- 16% of patients (35 ÷ 220 × 100) were rural

You can further demonstrate the inequality between women and men, and between rural and urban dwellers, by calculating the absolute gap (subtract the smaller number from the larger number) and the relative gap (divide the larger number by the smaller number).

**Table 2.** Measures of inequality: absolute gap and relative gap

	Absolute gap *Difference*	Relative gap *Ratio*
**Gender**	Men − Women 125 − 95 = **30**	Men ÷ Women 125 ÷ 95 = **1.3**
**Place of residence**	Urban − Rural 185 − 35 = **150**	Urban ÷ Rural 185 ÷ 35 = **5.3**

In a report, you would normally include Table 1 (which shows the number of operations in each subgroup) and compare the percentages in each subgroup). In addition, you can use the information about the relative ratio to point out that there were 1.3 times more male than female patients, and 5.3 times more urban than rural patients.

This information alerts you that rural dwellers, and rural women in particular (of whom there were only 4), are not accessing your services as much as their urban counterparts.

You then decide to do more outreach and deliver two outreach activities in the next quarter. The monitoring data for April to June is given in Table 3.

**Table 3.** Tally of cataract operations after outreach

	Women	Men	Total
**Rural**	35	41	76
**Urban**	62	59	121
**Total**	97	100	197

**Table 2.** Measures of inequality after outreach

	Absolute gap *Difference*	Relative gap *Ratio*
**Gender**	Men − Women 100 − 97 = **3**	Men ÷ Women 100 ÷ 97 = **1.0**
**Place of residence**	Urban – Rural 121 − 76 = **45**	Urban ÷ Rural 121 ÷ 76 = **1.6**

You might conclude from this that providing outreach services has been effective in reducing both gender and place of residence inequalities. You could use this information to advocate for regular outreach activities and continue to monitor your services each quarter to identify further changes that are needed.
